# Clinical Glycomics Employing Graphitized Carbon Liquid Chromatography–Mass Spectrometry

**DOI:** 10.1007/s10337-014-2813-7

**Published:** 2014-12-09

**Authors:** Kathrin Stavenhagen, Daniel Kolarich, Manfred Wuhrer

**Affiliations:** 1 Division of BioAnalytical Chemistry, VU University Amsterdam, De Boelelaan 1083, 1081 HV Amsterdam, The Netherlands; 2 Department of Biomolecular Systems, Max Planck Institute of Colloids and Interfaces, Wissenschaftspark Potsdam-Golm, Am Mühlenberg 1 OT Golm, 14242 Potsdam, Germany; 3 Department of Molecular Cell Biology and Immunology, VU University Medical Center, Amsterdam, The Netherlands; 4 Center for Proteomics and Metabolomics, Leiden University Medical Center, Leiden, The Netherlands

**Keywords:** Porous graphitized carbon, Mass spectrometry, N-glycans, O-glycans, Clinical glycomics, Glycopeptides

## Abstract

Glycoconjugates and free glycan are involved in a variety of biological processes such as cell–cell interaction and cell trafficking. Alterations in the complex glycosylation machinery have been correlated with various pathological processes including cancer progression and metastasis. Mass Spectrometry (MS) has evolved as one of the most powerful tools in glycomics and glycoproteomics and in combination with porous graphitized carbon–liquid chromatography (PGC–LC) it is a versatile and sensitive technique for the analysis of glycans and to some extent also glycopeptides. PGC–LC–ESI–MS analysis is characterized by a high isomer separation power enabling a specific glycan compound analysis on the level of individual structures. This allows the investigation of the biological relevance of particular glycan structures and glycan features. Consequently, this strategy is a very powerful technique suitable for clinical research, such as cancer biomarker discovery, as well as in-depth analysis of recombinant glycoproteins. In this review, we will focus on how PGC in conjunction with MS detection can deliver specific structural information for clinical research on protein-bound N-glycans and mucin-type O-glycans. In addition, we will briefly review PGC analysis approaches for glycopeptides, glycosaminoglycans (GAGs) and human milk oligosaccharides (HMOs). The presented applications cover systems that vary vastly with regard to complexity such as purified glycoproteins, cells, tissue or body fluids revealing specific glycosylation changes associated with various biological processes including cancer and inflammation.

## Introduction

Glycans, either in free form or attached to proteins and lipids, are important key molecules found on cellular surfaces, in the extracellular matrix and in secreted fluids. These glycans and glycoconjugates are involved in a variety of vital biological processes such as cell–cell and cell–host interaction as well as cellular trafficking. Alterations in the complex glycosylation machinery responsible for the biosynthesis of glycoconjugates have frequently been correlated with cancer progression and metastasis [1–4]. As a consequence, several established biomarkers for different types of cancer such as colorectal cancer (carcinoembryonic antigen—CEA), ovarian cancer (cancer antigen-125—CA-125) or prostate cancer (prostate-specific antigen—PSA) are glycoproteins or specific glycan epitopes [5]. Despite the fact that glycosylation changes in diseased tissues have already been discovered decades ago [6], sophisticated approaches to capture the glycome and glycoproteome of purified glycoproteins, cells, tissues or body fluids in a sensitive and selective manner have just recently been established, allowing to study these changes in detail at a molecular level.

Various glycoanalytical technological developments of recent years have enabled the detailed characterization of disease-associated glycosylation changes. Many of these technological advances have been realized by using mass spectrometry (MS) techniques, which have evolved as some of the most powerful tools for glycan and glycoconjugate analysis. Electrospray ionization (ESI)–MS and matrix-assisted laser desorption/ionization (MALDI)–MS are the most applied techniques for carbohydrate analysis, performed in positive and negative ionization mode [7]. MALDI–MS of carbohydrates in their native and also derivatized form, such as permethylation, is widely applied and has been comprehensively reviewed recently [8–14]. However, samples containing glycan isomers might be not distinguished by this approach. Various online separation techniques coupled to ESI–MS can overcome this issue. There are well-established approaches for glycan analysis using liquid chromatography (LC) and electrophoretic separation coupled to MS which have been compared with each other in several reviews, e.g., hydrophilic interaction–liquid chromatography (HILIC) of fluorescently labeled glycans, high-performance anion exchange chromatography (HPAEC) or porous graphitized carbon (PGC)–LC–ESI–MS of native and reduced glycans, as well as capillary electrophoresis (CE)–MS and capillary gel electrophoresis (CGE)–MS of native and derivatized glycans [15–20]. Recently, several multi-institutional studies evaluated some of these different techniques for the analysis of protein glycosylation [21–23].

PGC–LC in combination with ESI–MS/MS detection is a versatile and sensitive tool for the analysis of released and free glycans and, with some limitations, also glycoconjugates such as glycopeptides [24]. In this review, we will focus on how PGC-based approaches can deliver specific structural information on protein-bound N-glycans, mucin-type O-glycans and briefly also on glycopeptides, glycosaminoglycans (GAGs) and human milk oligosaccharides (HMOs).

A common approach for the analysis of protein N-glycosylation involves the enzymatic release of these glycans using peptide-N-glycosidase F (PNGase F). The enzyme releases the N-glycans by forming a glycosylamine intermediate which may convert into a glycan exhibiting a free reducing end. This enables the analysis of glycans either with a reducing end or after reduction as alditols. Another widely used strategy targets specifically the reducing end by chemical derivatization, where fluorescent labels such as 2-aminobenzamide or 9-aminopyrene-1,4,6-trisulfonic acid are added via reductive amination as reviewed before [18]. Since there is no enzyme that would allow a global release of O-glycans from the protein backbone, reductive β-elimination is the method of choice concomitantly releasing and reducing the O-glycans in a single-step procedure [25–28]. However, this technique does not allow subsequent labeling with a fluorescent tag and thus non-reductive, chemical release approaches are further investigated [29–33] ).

## Characteristic Features of PGC-Based Glycan Analysis

For the analysis of released glycans by PGC–LC, just minimal sample preparation is required, since no chemical derivatization of the glycan compounds is required such as labeling of the reducing end or permethylation. Thus, PGC–LC coupled to tandem mass spectrometry is almost exclusively performed on underivatized oligosaccharides in their reduced or non-reduced (native) form, which has the advantage that sample losses due to incomplete derivatization and additional purification steps can be minimized. This feature makes PGC–LC also one of the most widespread methods for the analysis of O-glycans released by reductive β-elimination [25, 26, 34].

PGC chromatography is frequently used in the solid-phase extraction (SPE) mode for oligosaccharide desalting as well as purification prior to MS analysis [35]. It has been successfully used for desalting and purification of N-glycans [25, 36–41], O-glycans [42–44], GAGs [45–47] and glycans derived from glycolipids [48]. Consequently, also free oligosaccharides [49], including HMOs from different sources were enriched and cleaned-up by PGC–SPE [50–52].

Due to the fact that glycans themselves cannot be detected by any optical detection methods in sufficient nano/picomolar sensitivity, coupling of PGC–LC with MS has developed as a powerful approach for detection and characterization of native and reduced glycans. Depending on the type of solvent used for separation, the released glycans are detected either as positively or negatively charged species. Many approaches use negative ionization as the preferred approach [24, 25], which results in fragmentation spectra that give rise to more specific cross-ring cleavages in MS/MS spectra, facilitating structure characterization [53–57]. Notably, signal intensities of acidic glycans detected in negative-ion mode can be more pronounced than those of the neutral glycans [58], and consequently correction factors can be introduced to allow accurate relative quantitation [59]. Nevertheless, when reduced N-glycans are analyzed in positive-ion mode, signal intensities of simultaneously analyzed acidic and neutral glycans tend to show ratios that are comparable to results obtained from HILIC separation with fluorescence detection of 2-AB-labeled glycans [58]. Labeling glycans with a chromophore followed by HILIC analysis with fluorescence detection is an alternative approach, resulting in higher sensitivity, but introduces also additional sample preparation steps [18].

**Fig. 1:**
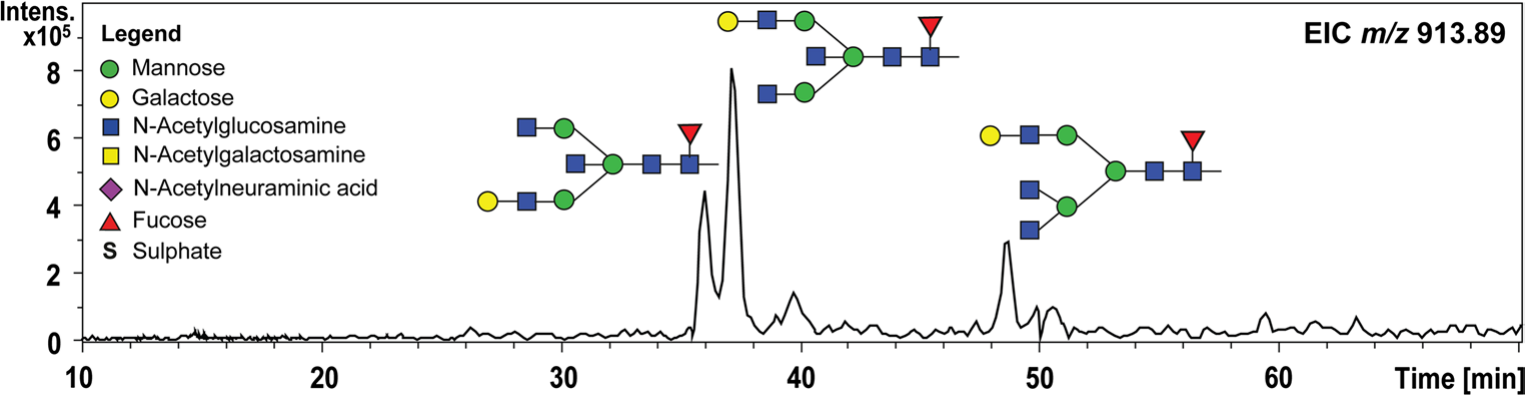
PGC–LC–ESI–IT–MS EICs of *m*/*z* 913.84, showing the different elution times of three N-glycan isomers with the composition Hexose_4_N-acetylhexosamine_4_Fucose_1_, derived from human colon tissue of an ulcerative colitis patient. Separation of the isobaric structures allows separate MS/MS analyses for in-depth structural characterization of the respective N-glycans. The EIC illustrates the different elution of structures with different glycan features, as the N-glycans containing a bisecting GlcNAc elute earlier than structural isomers with an additional antenna

**Fig. 2:**
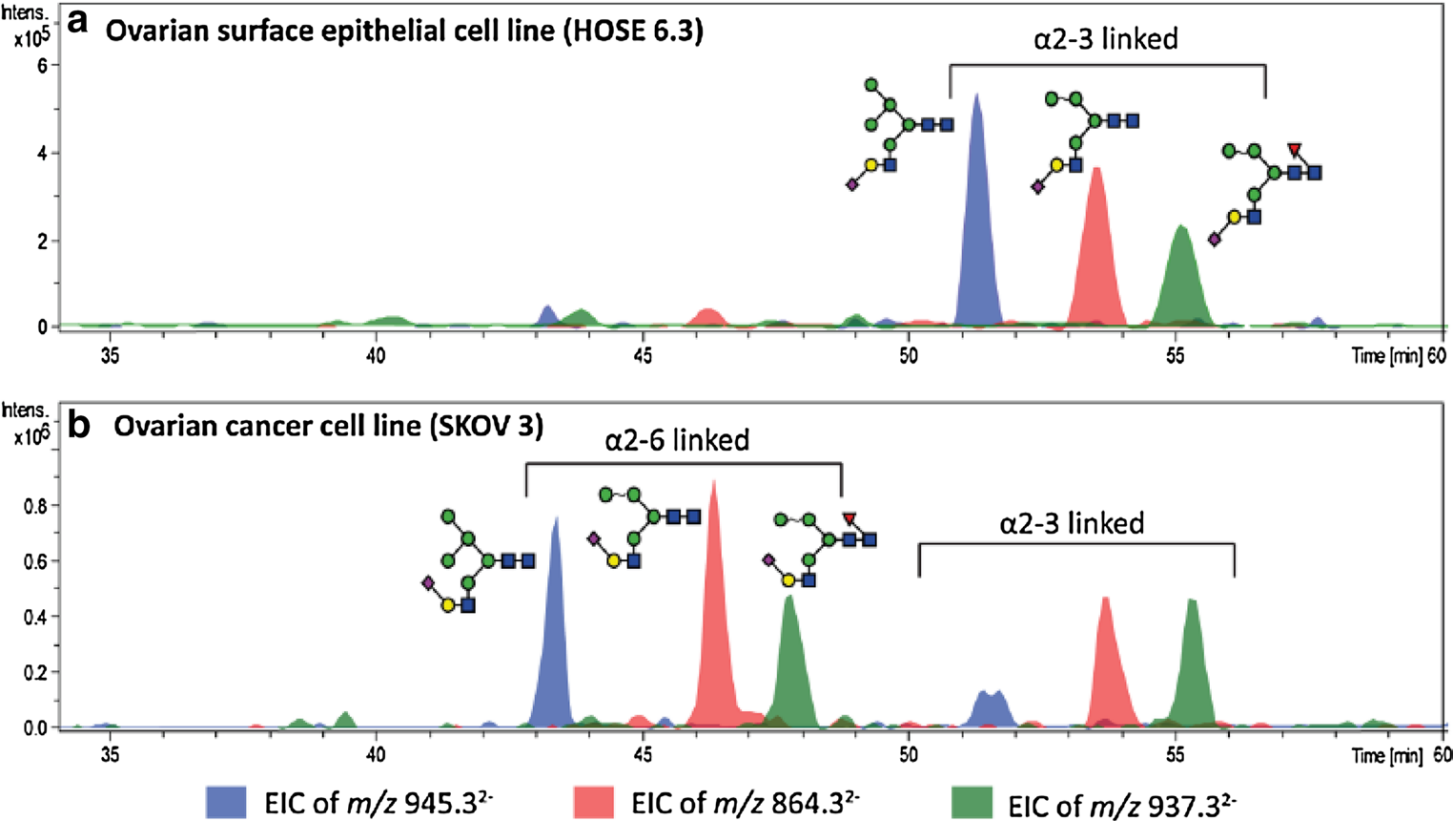
PGC–LC–ESI–IT–MS EICs of monosialylated hybrid N-glycans in **a** the non-cancerous epithelial cells (HOSE 6.3) and **b** ovarian cancer cell line (SKOV 3). The authors found a set of different N-glycan structures containing α(2,6)-linked sialic acid exclusively in ovarian cancer cell lines but not in non-cancerous cell lines as represented for HOSE 6.3 and SKOV 3. The EICs further illustrate the different retention behaviors of linkage isomers with α(2,6)-linked and α(2,3)-linked sialic acid, as α(2,3)-linked sialic acid containing glycans are stronger retained and elutes later in the gradient [61]. © 2014 American Society for Biochemistry and Molecular Biology

One of the most distinguished features of PGC–LC of glycans is the high separation power for structural and linkage isomers, which complements in particular MS analyses as compounds exhibiting exactly the same *m*/*z* can be separately analyzed. This feature makes PGC–LC–ESI–MS/MS a very capable tool for screening of disease specific glycosylation signatures. Particular structural features of glycans are known to influence the elution behavior in PGC chromatography, e.g., N-glycans carrying a bisecting N-acetylglucosamine (GlcNAc) are eluting several minutes earlier than their non-bisected structural isomers that carry an additional antenna (Fig. 1). The linkage of sialic acid residues has also been shown to alter the retention time behavior, with α(2,3)-linked structures eluting later compared to their α(2,6)-linked counterparts [19, 58, 60], which is also demonstrated for a set of hybrid sialo N-glycoforms in ovarian cancer cell lines in Fig. 2 [61]. This distinct feature of isomer separation provides valuable information in studies focusing on cancer glycosylation, as alterations in expression of α(2,6)-sialyltransferases and thus α(2,6)-sialylated glycans are associated with cancer progression [62]. The separation power of PGC is not limited to sialylated glycans but has also been successfully applied in the differentiation and characterization of fucosylated N- and O-glycans, resolving glycan isomers with LeX, LeA, LeY and LeB structural elements [44] as well as oligomannosidic N-glycans [63]. The high capacity of PGC to separate isomeric glycans thus makes it a perfect tool to be combined with MS detection for relative quantitation of single structural isomers and structural characterization. Besides PGC also other separation techniques are able to separate isomers to a certain extent, as reviewed elsewhere for HILIC [18, 20, 64–67], reversed phase (RP)–LC [15, 18], high-performance anion exchange chromatography (HPEAC) [68] and capillary as well as capillary gel electrophoresis (CE and CGE) [18, 20, 69], which are not detailed in this review. However, the isomer-selective separation of PGC cannot be reached by these methods, as previously shown in a systematic comparison of RP, HPAEC, HILIC and PGC [70] and as was reviewed for sialylated glycoforms [19].

Several publications have focused on the elucidation of N- and O-glycan fragmentation pathways of released glycans in negative-ion mode. The determined fragmentation patterns and diagnostic ions specific for different glycan features allow a detailed structural elucidation [26, 53–57, 71, 72]. Recently, it has been shown that glycan fragmentation is conserved for negatively charged precursors in ESI-ion trap(IT)-MS/MS, even if instruments from different vendors are used in different laboratories [73]. Thus, the collection of a large number of N- and O-glycan spectra in an open access database organized by the UniCarb-DB initiative [74] presents an important first step to facilitate data analysis using reference spectra and makes PGC–LC–ESI–MS/MS-based glycomics accessible to a broader audience of researchers.

## Applications of PGC–LC–ESI–MS in the Analysis of Disease-Associated Glycosylation Signatures

### N-Glycans

PGC–LC–ESI–MS/MS allows monitoring and detailed characterization of particular disease-associated N-glycosylation signatures. This allows the evaluation of individual glycan species data or further functional grouping (e.g., complex, hybrid, high-mannose glycans) and relative quantification of glycan features and epitopes such as Lewis-type and blood group epitopes, α(2,3)- and α(2,6)-linked sialic acids or bisecting GlcNAc structures. The comparative analysis of specific glycan features derived from control and disease samples pinpoints to alterations in the glycan biosynthesis such as differential expression of glycosyltransferases, providing important first hints for further investigations, which aim to understand onset and progression of a disease.

### N-Glycan Analysis of Cancer Cell Lines

The PGC–LC–ESI–MS/MS glycomics approach has been applied by the group of Nicolle Packer to investigate glycosylation changes occurring in different cancer cell lines such as colorectal cancer [75, 76], leukemia [77] and ovarian cancer [61]. N-glycans and subsequently O-glycans were released using a polyvinylidene fluoride (PVDF) membrane protein immobilization approach, followed by analysis of glycan alditols in negative-ion mode ESI-IT–MS/MS [25]. Manual structural elucidation of glycan fragmentation spectra and relative quantification based on the area under the curve (AUC) of the corresponding extracted ion chromatograms (EICs) was used to obtain the identity and the relative amount of the individual glycan components present in a particular sample.

A recent study on ovarian cancer compared the membrane N-glycome of two non-cancerous ovarian surface epithelial cell lines and four ovarian cancer cell lines with the gene expression of the corresponding key glycosyltransferases [61]. In total 70 individual N-glycan structures derived from 53 identified compositions were detected and their relative abundances determined. The cancer cell lines showed a larger portion of high-mannose glycans and a reduced amount of complex sialylated N-glycans. Additionally, a set of N-glycans carrying α(2,6)-linked sialic acid and bisecting GlcNAc as well as mono-, di-fucosylated, and sialylated LacdiNAc (N-acetylgalactosamine β(1,4)N-acetylglucosamine β1-) structures was exclusively found in the cancer cell lines and not in non-cancerous ovarian surface epithelial cell lines. In Fig. 2, two panels with representative EICs of hybrid sialo N-glycans are depicted that show the specific expression of α(2,6)-linked sialic acid containing structures in ovarian cancer cell lines compared to ovarian epithelial cell lines. A gene expression analysis of various glycosyltransferases including α(2,6) sialyltransferase (ST6GAL 1 gene), bisecting GlcNAc transferase (MGAT 3 gene), β(1,3/4) N-acetyl-galactosaminyltransferases (B3GALNT and B4GALNT3 genes), ST3Gal sialyltransferases (ST3GAL 1-5) and six α-(1,2/3/4/6) fucosyltransferases (FUT2-5,8,9) showed that gene expression of the ST6GAL 1, MGAT 3, and B4GALNT3 genes was increased, whereas the ST3GAL 5 gene expression was decreased in cancer cell lines. This data confirmed the direct correlation of gene expression and specific N-glycan changes occurring in the analyzed cell lines [61].

Sethi et al. compared the N-glycosylation of three different colorectal cancer cell lines classified as “moderately differentiated”, “moderately differentiated metastatic” and “poorly differentiated—aggressive” [76]. They detected 42 N-glycan structures derived from 34 different compositions containing a high proportion of high-mannose glycans as well as lower amounts of hybrid, complex and also paucimannosidic glycans. When the sialic acid containing glycans were grouped, they exhibited a different expression profile between the three cell lines analyzed. In particular, α(2,3)-linked sialic acid containing N-glycans were only found in the more aggressive cell line, while α(2,6)-linked sialic acid was present on N-glycans in all samples, indicating a correlation between sialic acid expression and tumor progression. Furthermore, several N-glycans containing a bisecting GlcNAc were exclusively detected in the metastatic cell line. Orthogonal confirmation of these results was obtained using the bisecting GlcNAc-recognizing PHA-E lectin and mRNA expression level analysis of the Mgat3 gene, which encodes the bisecting GlcNAc transferring GlcNAc transferase III [76].

A recent study by Chik et al. [75] compared the glycosylation profiles of four colorectal cancer cell lines with the ones obtained from human colorectal tumors and found distinct glycan differences in the tissue samples compared to the cell line-derived samples. In total 173 N-glycan and 43 O-glycan structures were detected and confirmed by manual annotation of fragmentation spectra and their relative abundances were quantified. The expression of different glycans in the cell lines compared to the tumor epithelial tissue may indicate that cell surface molecule glycosylation is adapted to cell culture conditions over time in established cell lines. This work clearly indicates that the aspect of protein glycosylation needs to be carefully considered when planning and performing cell line-based biomarker discovery studies or when evaluating or reassessing tissue-derived glycomics information within cancer cell lines [75].

### Analysis of N-Glycans Derived From Cancer Patient Human Plasma

Besides reduced N-glycans also non-reduced N-glycans are commonly analyzed on PGC–LC. The analysis of non-reduced glycans results in an additional separation of alpha and beta anomers, which introduces an additional level of complexity [78] that is caused by the PNGase F release: On the protein, the N-glycan is exclusively attached in beta configuration to the asparagine side chain while for released N-glycans a spontaneous conversion of alpha and beta anomers takes place resulting in an equilibrium. The group of Carlito Lebrilla is using this approach for biomarker studies in different cancer types by analyzing human plasma N-glycans [79–81]. The authors use a microfluidic chip including a PGC guard and analytical column [82] which is mostly combined with an additional PGC–SPE clean-up step prior mass spectrometric analysis. This chip-based approach is featuring a highly reproducible chromatography, since unstable absolute retention times can cause problems in PGC–LC data analysis [83]. The released N-glycans were detected as positively charged species, also because an acidic LC-buffer system consisting of 0.1 % formic acid in water (solvent A) and 0.1 % formic acid in acetonitrile (solvent B) was used. In this context, it needs to be mentioned that ionic strength and pH in different LC-buffer systems have an effect on the glycan recovery, which has been systematically investigated recently by Pabst and Altmann [58]. They reported a poor recovery of highly sialylated structures in unbuffered systems with low ionic strength. This needs to be considered carefully, since reports using the 0.1 % formic acid in water and acetonitrile solvent system are observing a loss of higher sialylated glycans [84].

Ruhaak et al. compared different protein enrichment techniques from human plasma of 20 lung cancer patients and 20 control persons for a lung cancer glycan biomarker discovery study [81]. N-glycan profiles of whole plasma, enriched IgG, enriched medium abundance proteins and their corresponding wash fractions were analyzed. For statistical evaluation, relative intensities of structural isomers were combined into single glycan compositions. In addition, these compositions (between 79 and 20 depending on the fraction) were grouped according to general glycan features such as high-mannose, complex or hybrid (C/H) non-sialylated and non-fucosylated, C/H fucosylated, C/H sialylated and C/H fucosylated and sialylated glycans. Data analysis revealed significant differences in relative intensities of glycan compositions and features between cancer patients and control persons for the IgG-enriched fraction and whole plasma, but not for medium abundance proteins. These results suggest that glycosylation differences in plasma of lung cancer patients are more pronounced on higher abundant proteins [81].

In a different study from the Lebrilla group serum N-glycans were analyzed from a large cohort of ovarian cancer patients (*n* = 199) and control subjects (*n* = 100) to investigate potential glycan biomarkers [80]. Their statistical analysis of glycan profiles was performed in 2 steps: First, a model was developed based on a training set, which was then used for classification of a second set of samples. They found the differential expression of a set of 22 glycan compositions in three different tumor stages when compared to the control samples. From these 22 glycan compositions, two were being expressed in higher and 20 of them in lower abundance in tumor patient sera. They could also show that a combination of up to nine glycan compositions, on an individual level as well as on a group level, could be used for classification between cancer and control cases [80].

### O-Glycans

PGC–LC has widely been applied in the analysis of O-glycans, in particular derived from mucins. These mucin-type O-glycans, which are generally attached via an O-GalNAc to a serine or threonine residue, are largely found in mucous membranes on secreted gel-forming mucins, including MUC2, MUC5AC, MUC5B, MUC6, MUC7 and MUC19, or on membrane-bound cell surface mucins such as MUC1, MUC3A, MUC3B and MUC4 [85]. However, mucin-type O-glycans can be also attached to non-mucin glycoproteins such as plasma glycoproteins [86, 87]. Changes in mucin-type O-glycosylation have also been associated with inflammatory diseases and cancer [88, 89]. With the help of PGC–LC–ESI–MS/MS mucin-type O-glycans were analyzed from different body fluids and tissues [42, 90–93], investigating their role in pathogen binding [44, 94, 95], and with respect to several diseases such as rheumatoid arthritis [96, 97], cystic fibrosis [98, 99] and ovarian cancer [100]. The method of choice to release O-glycans from the glycoproteins in all studies discussed in this review was reductive β-elimination prior PGC–LC–ESI–MS/MS analysis [25].

**Fig. 3:**
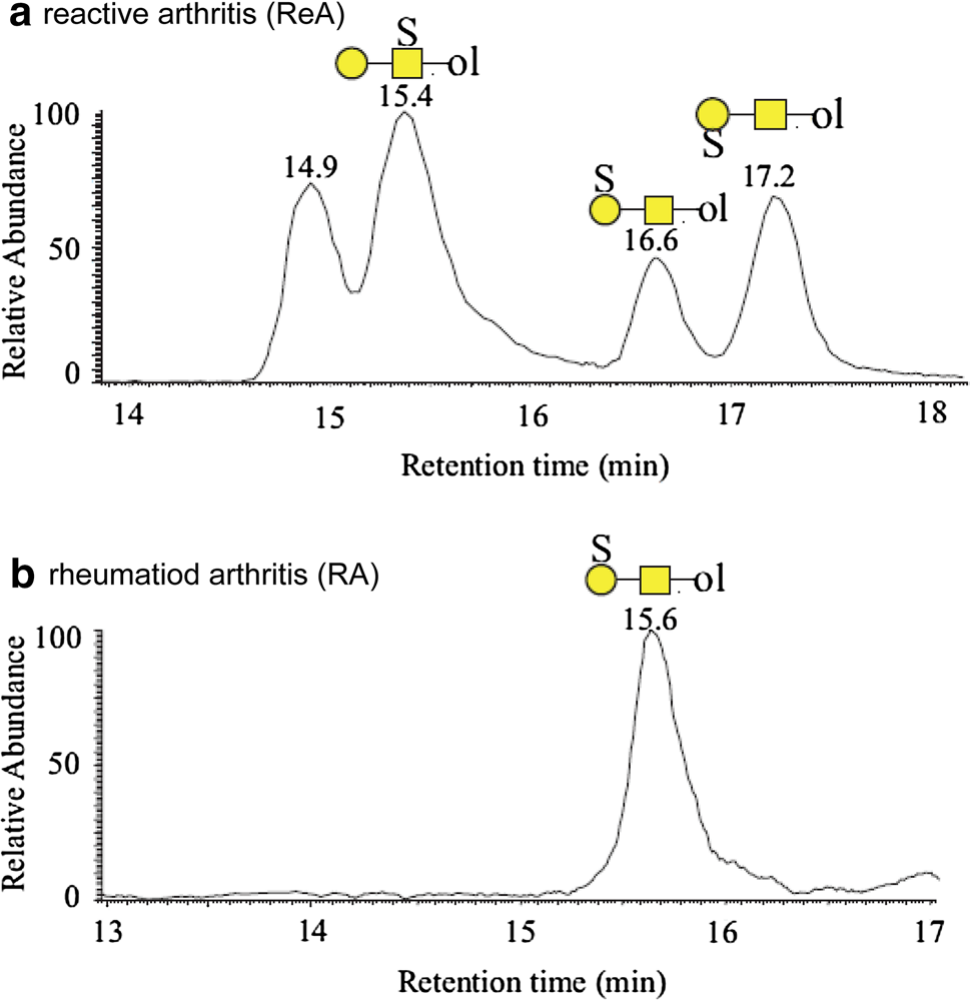
PGC–LC–ESI–IT–MS EICs of sulfated core 1 O-glycan isomers (*m*/*z* 464.1) from acidic glycoproteins of synovial fluid from a patient with ReA (**a**) and RA (**b**). ReA patients showed a more diverse isomer pattern as it contains one structure with the sulfate linked to the GalNAc (RT 15.4 min) and two structures with a Gal-linked sulfate (RT 16.6 and 17.2 min). In contrast RA patients carry just a single Gal-linked sulfate structure on their synovial acidic glycoproteins (RT 15.6 min) [96]. © 2014 American Society for Biochemistry and Molecular Biology

The group of Niclas Karlsson published several disease-related O-glycan studies such as the investigation of lubricin O-glycosylation derived from synovial fluids from rheumatoid arthritis (RA) patients. Specific sulfotransferases are known to be expressed in inflamed synovial fluids (SF) of RA patients [101] and the O-glycan characterization of lubricin, the major component of the acidic protein fraction in SF, revealed mainly mono- and disialylated core 1 and core 2 structures, as well as sulfated core 2 structures [97]. In a follow-up study, they analyzed the acidic protein O-glycosylation in SF of RA patients and found distinct differences in glycan expression in the acute form (reactive arthritis—ReA) of the disease compared to the chronic RA. ReA SF contained three structural and linkage isomers of the sulfated core 1 glycan (Galβ1–3GalNAcol) (Fig. 3a), whereas in the SF of chronic RA patients just one isomer could be detected (Fig. 3b) [96]. Since RA is a systemic disease glycosylation changes can also be expected to be present on a global level in addition to changes occurring at the site of inflammation. Consequently, O-glycosylation of salivary MUC7 in RA patients (*n* = 10) was analyzed and compared to control samples (*n* = 11) with regard to the sulfated core 1 O-glycan using a glycan-specific selected reaction monitoring (SRM) approach developed by the Karlsson group. With this sensitive and selective method, the sulfated 3-Gal-linked isomer of the core 1 glycan (Galβ1-3GalNAcol) could be quantified relative to the non-sulfated counterpart. They found a significantly higher expression of the sulfated form in RA patients (37.2 ± 3.11 %) compared to the control group (25.5 ± 1.75 %) showing that in the case of RA a change of glycosylation occurs at the site of inflammation and on a systemic level [96]. This particular SRM approach for the relative quantification of specific compounds benefits significantly from the isomer separation power of PGC chromatography.

**Fig. 4:**
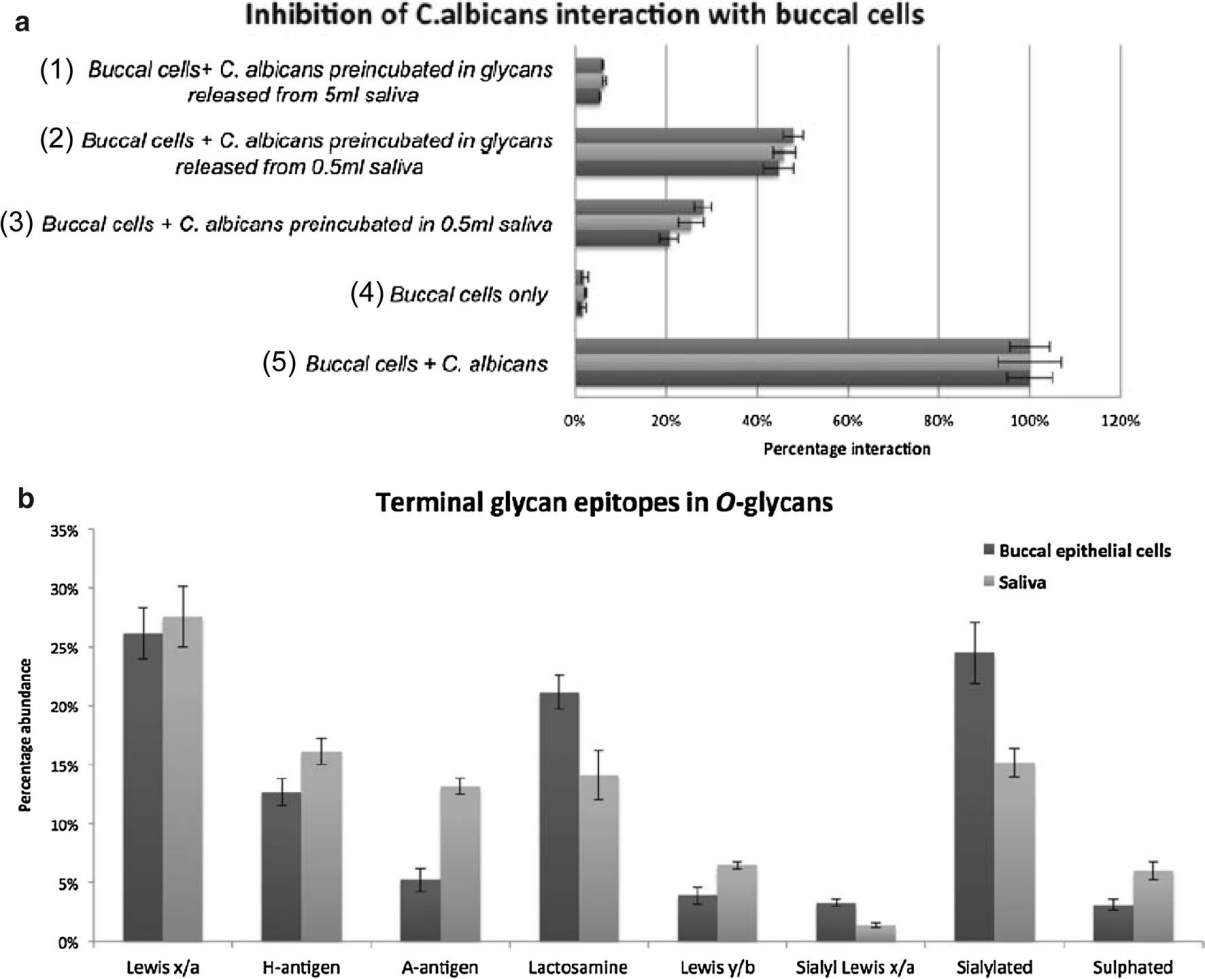
Flow cytometry-based assay to quantify the adhesion of *Candida albicans* to BEC. *a5* shows the adhesion of *C. albicans* to BEC normalized to 100 % and *a4* the corresponding autofluorescence of BEC only. The inhibition of interaction was analyzed after incubation with 0.5 mL of whole saliva *a3*, N- and O-glycans released from 0.5 mL saliva *a2* and N- and O-glycans released from 5 mL saliva *a1*. Salivary glycans as well as whole saliva inhibits binding of *C. albicans* to BEC. The graph contains the mean ± standard error of three independent biological replicates and their technical triplicates. (**b**) shows relative intensities of glycan epitopes from O-glycans of salivary and BEC membrane proteins, which express similar glycan epitopes in different relative intensities. Relative quantification was performed on MS ion intensities of all glycans carrying these epitopes. The graph contains the mean ± standard error of three technical replicates of both saliva and BEC collected from an individual of blood group A secretor status [44]. © Oxford University Press

A different study by Everest-Dass et al. [44] focused on the potential role of N- and O-glycosylation in infection. First, they provided a comprehensive inventory of the N- and O-glycosylation present in human saliva and on buccal epithelial cells (BEC) using PGC–LC–ESI–MS/MS, identifying 78 N-glycan and 112 unique O-glycan structures. The resolving power of PGC chromatography and structural elucidation by manual glycan spectra interpretation allowed a detailed comparison of specific glycan epitopes present in saliva and on buccal epithelial cells, including different Lewis and blood group epitopes, which are known receptors for pathogen adhesion [102]. Overall, Everest-Dass et al. [44] found similar glycan epitopes with differing relative intensities in both saliva and on buccal epithelial cells (Fig. 4b). They further investigated the potential of salivary protein glycosylation to inhibit *Candida albicans* infection. A flow cytometry-based cell adhesion assay confirmed that salivary glycans in different amounts as well as whole saliva inhibit binding of *Candida albicans* to buccal epithelial cells [Fig. 4a (panel 1–5)]. These data indicate a role of saliva glycans in mimicking epithelial cell epitopes and inhibiting pathogen binding as part of a first immune defense [44].

## The Application of PGC Chromatography for the In-depth Characterization of Glycoproteins and Glycopeptides

Besides using PGC–LC–ESI–MS/MS as a tool for the analysis of complex glycan mixtures it is also applied as an important tool for the in-depth characterization of glycoproteins. Detailed elucidation of the protein-specific types of glycans, their structures and compositions represents an important pillar in the comprehensive analysis of glycoprotein micro- and macroheterogeneity.

Deshpande et al. used PGC–LC–ESI–MS/MS to analyze the protein-specific glycosylation patterns of the four major protein components of secretory IgA (secretory component, IgA1&IgA2 and joining chain) [103]. Distinct glycan profiles of these proteins were determined, clearly showing that the secretory component carries mostly neutral, LeX-containing N-glycans, whereas neutral, bisected N-glycans and core 1 and core 2 type O-glycans were the dominating structures on IgA. In contrast, the joining chain protein carried mostly mono- and disialylated N-glycans with and without core fucose [103]. With this information the respective site-specific glycosylation features were determined by RP–LC–ESI–MS/MS of glycopeptides showing that every site in all of the glycoproteins carries a specific set of glycans. A similar approach has also been applied in studying human and recombinant IgGs [104], human butyrylcholinesterase [105] and recombinant human follicle-stimulating hormone [41].

**Fig. 5:**
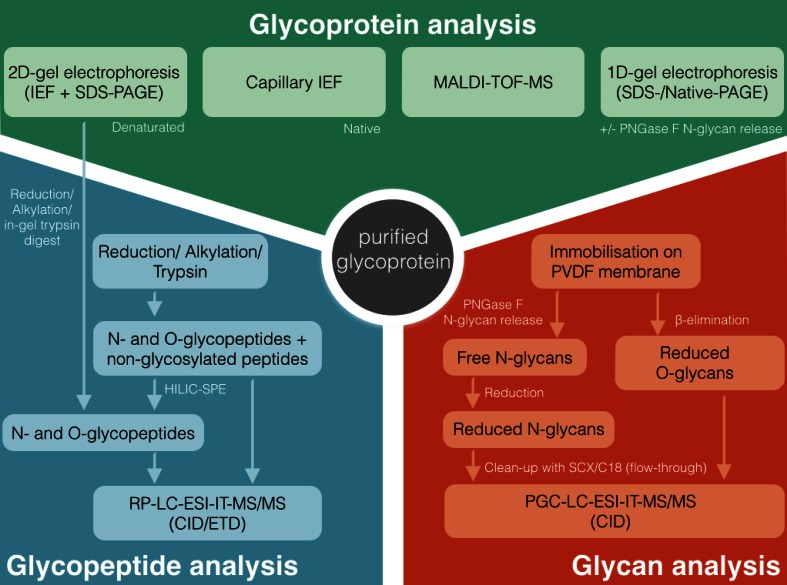
A representative workflow for a multi-experimental comprehensive characterization of a purified glycoprotein that shows the suitability of PGC–LC–ESI–MS/MS implementation into an analytical workflow. The glycoprotein is analyzed on three different levels, including the analysis of the intact (glyco)protein in a top-down approach (*upper part*). Besides that glycans are characterized in a bottom-up approach by blotting the protein on a PDVF membrane and subsequent release of N- and O-glycans, which are then analyzed on PGC–LC–ESI–MS/MS (*lower right part*). This information is then be used for peptide and glycopeptide characterization in a bottom-up approach to elucidate the full micro- and macroheterogeneity of the glycoprotein (*lower left part*). Modified from Sumer-Bayraktar et al. [106]

Another elegant approach for the in-depth characterization of a single, purified glycoprotein is shown in the representative analytical workflow depicted in Fig. 5 that was applied for the analysis of a purified plasma glycoprotein [106], where the authors combined a variety of different techniques to achieve a comprehensive characterization of the glycoprotein. Besides top-down approaches on the intact glycoprotein, e.g., MALDI–TOF–MS and 1D/2D gel electrophoresis with and without the use of specific glycosidases also PGC–LC–ESI–MS was integrated into the workflow as part of a bottom-up approach to elucidate the glycan moieties attached to the protein. The obtained glycan information was then used for site-specific glycosylation analysis on the glycopeptide level using RP–LC–ESI–MS/MS to gain further information about which specific N- and O-glycan species are attached to which glycosylation site.

PGC–LC–ESI–MS can also be applied for the analysis of intact glycopeptides [107–112]. However, it needs to be considered that the hydrophobicity of a glycopeptide increases with increasing peptide length, leading to a stronger interaction with the PGC stationary phase [107, 108]. To avoid the loss of (glyco-)peptides during PGC chromatography or SPE clean-up due to irreversible binding and low recovery, the peptide moiety should be kept as small as possible, but still in an appropriate length to obtain sufficient information on the peptide identity for unambiguous glycosylation site assignment. Unspecific or broad-specificity proteases, such as proteinase K or pronase, can be used for the enzymatic cleavage of glycoproteins resulting in small amino acid stretches that remain linked to the glycan. These “peptide tags” on the glycans have been described to be mostly sufficient for qualitative site-specific glycosylation analysis [109, 110]. Despite the presence of a short peptide tag, it needs to be carefully considered that highly sialylated glycopeptides generated by this approach may be retained irreversibly by the PGC column because of an increased interaction with the stationary phase, and thus just partial site-specific glycoprofiles would be obtained, with an expected bias towards low sialylation.

## PGC–LC for the Analysis of Glycosaminoglycans (GAGs)

GAGs such as heparan sulfate proteoglycans, for example, are involved in several processes such as basement membrane organization, cell signaling and morphogenesis [113]. Consequently, alterations in heparan sulfate glycosaminoglycans have been identified in several diseases [114]. In contrast to the above-discussed N- and O-glycans, GAGs are linear molecules consisting of repeating disaccharide units of hexosamines and hexuronic acids. The overall size of such GAG chains requires that they are digested by specific enzymes (e.g., keratanase, heparitinase or chondroitinase) into their disaccharide units, which are then commonly analyzed using LC–ESI–MS/MS (for further reading the detailed reviews by Joseph Zaia are recommended, comprehensively describing various approaches for MS-based GAG analyses) [17, 115]. PGC–LC–ESI–MS/MS is well suited for the analysis of disaccharides generated from GAGs [45, 116, 117] and has found numerous applications in the analysis of GAGs from plasma and serum [46, 47, 118, 119]. In a study by Wei et al., a method was developed and applied to analyze disaccharides from heparan sulfate (HS) GAGs in human serum using PGC–LC–ESI–MS/MS in negative-ion mode, providing compositional information on 12 disaccharides including structural isomers [46]. In a different study, by the same group free HS and heparan sulfate proteoglycans (HSPG) were analyzed from serum of 26 premenopausal and 25 postmenopausal women using PGC–LC–ESI–MS/MS [47]. Statistical analysis of the 12 HS-derived disaccharides revealed differences in four structures from HSPG and two structures from HS, including N-acetylated and N-sulfated disaccharides. These results indicated changes in the enzyme regulation of N-deacetylase/N-sulfotransferase in the context of menopause [47].

## Human milk oligosaccharides (HMOs)

PGC–LC has also been successfully applied in the analysis of HMOs, which are free sugars in milk of lactating women and present in concentrations between 5 and 20 g/L [120]. They are considered to have prebiotic effects to bacteria like *Bifidobacterium bifidum* and also to prevent pathogen binding to the intestinal mucosa by acting as analogs to cell surface epitopes [121]. In vitro studies have shown systemic, immunomodulatory effects of HMOs [122], which is also supported by their presence in urine [51], as well as in plasma of breastfed infants [52, 123]. Further biological functions of HMOs have been reviewed extensively elsewhere [124–126].

The analysis of HMOs by mass spectrometry has been discussed in detail by different groups, in part, also with respect to the use of PGC as stationary phase for LC separation. Similar to the glycan species discussed above, mainly HMOs with a free reducing ends were analyzed using PGC–LC [127–129].

Alpha-(1,2)-Fucosylated structures are found in blood group antigen secreting humans, specifically in their body fluids, including milk. Milk of secreting women, that contains high levels of α-(1,2)-fucosyloligosaccharides, has been associated with protecting full-term infants from diarrhea [130]. Using PGC–LC–ESI–MS α-(1,2)-linked fucosylation can be clearly distinguished from α-(1,3)-fucosylation by the different retention times of these isomers. Thus, a PGC nano-HPLC chip/TOF–MS approach provided a suitable analysis platform to compare HMOs from milk of mothers giving birth to full-term infants compared to preterm delivering mothers [131], since preterm infants are considered to exhibit an immature immune system [132]. The authors observed a large variation in the relative abundance of fucosylated structures in the milk derived from preterm delivering mothers. This example also emphasizes the importance of studies focusing on the analysis of specific fucosylation features [131].

## Conclusions and Future Perspectives

Recent technological developments made way for faster sample preparation as well as more sensitive and selective mass spectrometric analyses in glycomics and glycoproteomics. In this regard, porous graphitized carbon is a versatile and powerful tool, which has found a wide range of applications as SPE material and as a stationary phase for PGC–LC–ESI–MS applications for the analysis of predominantly native and reduced glycans. PGC features a high isomer separation, which in combination with tandem mass spectrometric analyses offers unique opportunities for specific glycan compound analyses of individual structures with high sensitivity and the seamless integration into already existing proteomics workflows. This capacity allows that functional questions regarding the role of specific glycan structures and features can now be addressed in a more protein-specific way. Consequently, this strategy becomes a more reliable technique also for clinical research such as cancer biomarker discovery and detailed characterization of therapeutic recombinant glycoproteins.

As a result of the isomer separation capacity of PGC–LC, data complexity can be already considerably high for glycan analyses of purified glycoproteins. To date, data analysis still represents a major bottleneck in glycomic analysis and needs to be addressed appropriately by (partial) automation to make PGC–LC–ESI–MS/MS-based glycomics approaches available and attractive to a broader scientific audience. One step towards this direction is the presence of several glycan databases containing experimental glycan fragmentation spectra [133]. Unicarb-DB contains a large selection of fragment spectra, which significantly facilitates the analysis of negative-ion mode PGC–LC–ESI–MS/MS data of native reduced N- and O-glycans. This database allows manual spectra matching with acquired data and is also meant to be used for automated structural assignment in the future [74]. Additionally, several research groups are focusing on developing software tools for compositional N-glycan identification on MS level and/or based on retention times, as well as tools that match acquired tandem MS spectra with theoretical fragmentation spectra [134–137]. Nevertheless, to fully exploit the unique potential for glycoconjugate analysis offered by PGC–LC–ESI–MS/MS-based approaches, concerted future efforts will be necessary to facilitate confident and automated glycan identification and quantification while maintaining sufficient data quality and reducing false positive assignments [138, 139].
